# A class III WRKY transcription factor in sugarcane was involved in biotic and abiotic stress responses

**DOI:** 10.1038/s41598-020-78007-9

**Published:** 2020-12-01

**Authors:** Dongjiao Wang, Ling Wang, Weihua Su, Yongjuan Ren, Chuihuai You, Chang Zhang, Youxiong Que, Yachun Su

**Affiliations:** 1 Key Laboratory of Sugarcane Biology and Genetic Breeding, Ministry of Agriculture, College of Agriculture, Fujian Agriculture and Forestry University, Fuzhou, 350002 Fujian China; 2 College of Life Sciences, Fujian Agriculture and Forestry University, Fuzhou, 350002 Fujian China; 3 Key Laboratory of Genetics, Breeding and Multiple Utilization of Crops, Ministry of Education, College of Agriculture, Fujian Agriculture and Forestry University, Fuzhou, 350002 Fujian China

**Keywords:** Molecular biology, Plant sciences

## Abstract

WRKY transcription factors play significant roles in plant stress responses. In this study, a class III *WRKY* gene *ScWRKY5*, was successfully isolated from sugarcane variety ROC22. The ScWRKY5 was a nucleus protein with transcriptional activation activity. The *ScWRKY5* gene was constitutively expressed in all the sugarcane tissues, with the highest expression level in the stem epidermis and the lowest in the root. After inoculation with *Sporisorium scitamineum* for 1 d, the expression level of *ScWRKY5* was significantly increased in two smut-resistant varieties (YZ01-1413 and LC05-136), while it was decreased in three smut-susceptible varieties (ROC22, YZ03-103, and FN40). Besides, the expression level of *ScWRKY5* was increased by the plant hormones salicylic acid (SA) and abscisic acid (ABA), as well as the abiotic factors polyethylene glycol (PEG) and sodium chloride (NaCl). Transient overexpression of the *ScWRKY5* gene enhanced the resistance of *Nicotiana benthamiana* to the tobacco bacterial pathogen *Ralstonia solanacearum*, however the transiently overexpressed *N. benthamiana* was more sensitive to the tobacco fungal pathogen *Fusarium solani* var. *coeruleum.* These results provide a reference for further research on the resistance function of sugarcane *WRKY* genes.

## Introduction

In the process of growth and development, plants often suffer from various external environmental challenges, such as drought, high salinity, cold, and pathogen stresses. After receiving stress signals in adversity, a complex regulatory network is formed in plants. Through the expression changes of various stress-related functional genes, the transcripts are recombined in plants to enhance adaptability to the environment. These gene expression changes and modifications are mainly regulated by transcription factors^1,2^. At present, several plant stress-related transcription factors have been reported, such as WRKY [Trp(W)-Arg(R)-Lys(K)-Try(Y), tryptophan-arginine-lysine-tyrosine]^3^, MYB (myeloblastosis)^4^, NAC [also call as NAM, ATAF1(2), CUC2]^5^, bZIP (basic leucine-zipper)^6^, AP2/ERF (APETALA 2/ethylene-responsive element binding factor)^7^, C2H2 zinc finger protein^8^, SPL (*SQUAMOSA* promoter-binding protein-like)^9^, DREB (dehydration responsive element binding protein)^10^, and CDFs (cycling DOF factors)^11^.

WRKY is considered to be a plant-specific transcriptional regulator^12^. Since the first sweet potato *WRKY* gene *SPF1* (SWEET POTATO FACTORS1) was cloned in 1994^13^, a large number of *WRKY* family genes have been gradually isolated and identified from various plants. There are 72 *WRKY* genes in the model plant *Arabidopsis thaliana*^3^, 105 *WRKYs* in *Oryza sativa*^14^, 68 *WRKYs* in *Sorghum bicolor*^15^, 45 *WRKYs* in *Hordeum vulgare*^16^, 119 *WRKYs* in *Zea mays*^17^, and 105 *WRKYs* in *Setaria italica*^18^. The most typical feature of WRKY transcription factors is that there is a polypeptide sequence of at least 60 amino acids in length in the DNA-binding domain of WRKY family members. A highly conserved heptapeptide sequence WRKYGQK occurs near the N-terminus of WRKY, which is an important criterion for identifying the WRKY family members^12^. A zinc-finger-like motif is found in the WRKY domin^12^. Based on the number of WRKY domains and the characteristics of the zinc-finger-like motif, the WRKY family can be divided into three types. Type I WRKYs contain two WRKY domains and a zinc-finger-like motif C_2_H_2_ (CX_4-5_CX_22 -23_HX_1_H) (X represents any amino acid residue). The WRKY domain at the C-terminus of type I WRKYs mainly mediates the binding of the protein to the target DNA, while the function of the N-terminal WRKY domain remains to be studied. Type II WRKYs contain only one WRKY domain, and their zinc-finger-like motif is the same as the type I WRKYs. According to the homology between family members, type II WRKYs can be further divided into five subclasses (IIa–IIe)^12^, while, Dong et al.^19^ divided the type II WRKYs of *A. thaliana* into seven subclasses (IIa–IIg). Only one WRKY domain is contained in type III WRKYs, and the zinc-finger-like motif C_2_HC (CX_7_CX_23_HX_1_C) differs from that of the type I and type II WRKYs^20^. Based on amino acid sequence similarity, 97 WRKY proteins in *O. sativa* were divided into three types and 13 groups, of which class II WRKYs were divided into 10 subclasses (IIa–IIj), and class III WRKYs were divided into two subclasses (IIIa and IIIb)^21^.

WRKY transcription factors play an important role in the response to biotic and abiotic stresses. In 1996, Rushton et al.^22^ found that *WRKY1*, *WRKY2*, and *WRKY3* in *Petroselinum crispum* have regulatory roles in the *PR1* gene-mediated immune response. Forty-nine of the 72 *WRKY* genes in *A. thaliana* were induced by *Pseudomonas syringae* and salicylic acid (SA) stresses^19^. However, the expression profile of *WRKYs* in *H. vulgare*, namely, *HvWRKY1* and *HvWRKY2*, was found to negatively regulate the resistance of *H. vulgare* to powdery mildew^23^. In *O. sativa*, 15 *WRKYs* were induced by *Magnaporthe grisea* infection, of which 12 could also be simultaneously induced by *Xanthomonas oryzae* pv. *oryzae*^24^. There were 88 *WRKYs* in *Phaseolus vulgaris* response to drought stress, among which eight were up-regulated and 11 were down-regulated^25^. Transcriptome analysis showed that of the 100 *WRKYs* in *Populus*, 61 were induced by *Marssonina brunnea*, SA, methyl jasmonate (MeJA), injury, cold, and salt stresses^26^. In maize, 58 *WRKY*s were induced by drought stress^27^.

Sugarcane (*Saccharum* spp.) is an important sugar crop globally^28,29^. Liu et al.^30^ found that the expression level of the class II sugarcane *Sc-WRKY* gene (GenBank Accession No. GQ246458) was induced by the sugarcane smut pathogen (*Sporisorium scitamineum*), SA, sodium chloride (NaCl), and polyethylene glycol (PEG), suggesting that the *Sc-WRKY* gene may play a role in the response mechanism of sugarcane to *S. scitamineum*, drought, and high salt stresses. Wang et al.^31^ showed that the expression level of class IIc sugarcane *ScWRKY3* gene (GenBank Accession No. MK034706) was decreased in the smut-susceptible *Saccharum* hybrid variety ROC22 and remained unchanged in the smut-resistant *Saccharum* hybrid variety Yacheng05-179. Additionally, the expression level of *ScWRKY3* was increased by NaCl, PEG, and the plant hormone abscisic acid (ABA), but was decreased by SA and MeJA. In contrast, Wang et al.^32^ indicated that the expression level of class IIc sugarcane *ScWRKY4* gene (GenBank Accession No. MG852087) was repressed in the Yacheng05-179 and remained unchanged in ROC22 after inoculation with *S. scitamineum*. The expression level of *ScWRKY4* gene was increased under ABA, SA, MeJA, NaCl, and PEG stresses. Zhang et at.^33^ found that the expression level of class IId sugarcane *ScWRKY6* gene (GenBank Accession No. MH393927) was increased under NaCl, PEG, and MeJA stresses. Li et al.^34^ showed that the expression profile of *SsWRKY* in different *Saccharum spontaneum* samples at 46 different developmental stages had different spatial and temporal patterns, with 52 *SsWRKY* genes expressed in all of the tissues, four *SsWRKY* genes not expressed in any tissues, and 21 *SsWRKY* genes possibly involved in photosynthesis. These above researches indicate that sugarcane WRKY family members of different types may have various functions due to their expression characteristics. In this study, a new class III *WRKY* gene, *ScWRKY5*, was successfully cloned from sugarcane variety ROC22. The sequence characteristics, subcellular localization, transcriptional self-activation activity, tissue-specific expression, gene expression patterns under different stresses, and transient expression of *ScWRKY5* in *Nicotiana benthamiana* after inoculation with the tobacco pathogens *Ralstonia solanacearum* and *Fusarium solani* var. *coeruleum* were analyzed.

## Results

### Cloning and sequence analysis of the *ScWRKY5* gene

**Figure 1:**
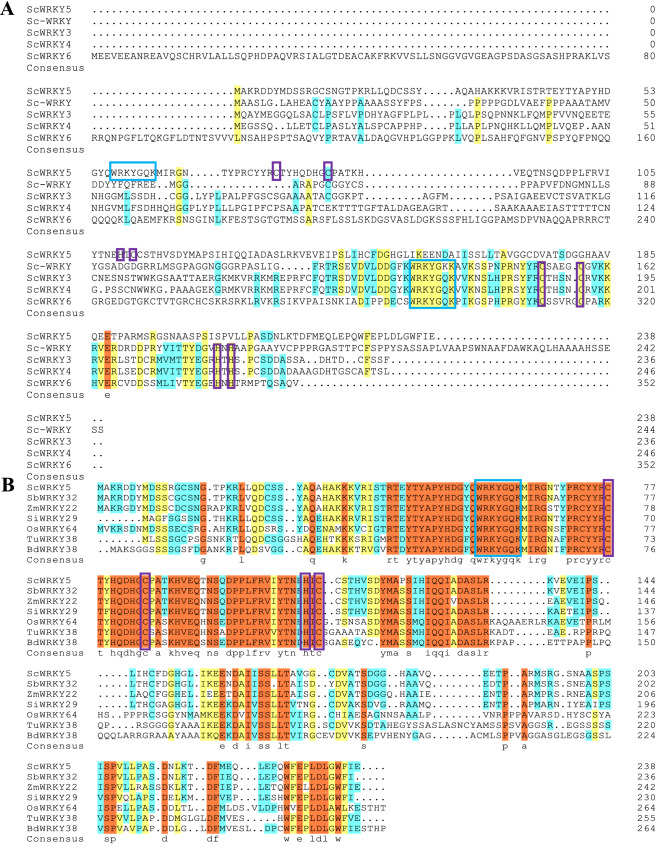
Protein sequence alignment of ScWRKY5 and WRKYs from sugarcane and other plant species by DNAMAN (version 6.0.3.99, Lynnon Biosoft) software. (**A**) The amino acid sequences of Sc-WRKY (ACT53875.1), ScWRKY3 (AYH64982.1), ScWRKY4 (AUV50355.1), ScWRKY6 (AXY87953.1) were identified from sugarcane. (**B**) The amino acid sequences of *Sorghum bicolor* SbWRKY32 (XP_002456546.1), *Zea mays* ZmWRKY22 (NP_001147816.1), *Setaria italica* SiWRKY29 (XP_004972233.1), *Oryza sativa* OsWRKY64 (XP_015642042.1), *Triticum urartu* TuWRKY38 (EMS45972.1), and *Brachypodium distachyon* BdWRKY38 (XP_010233534.1) were obtained from GenBank. The orange, yellow, blue, and white colors indicate the homology level of the conservation of the amino acid residues in the alignment at 100, ≥ 75, ≥ 50, and < 50%, respectively. The sequences of the WRKY motif (WRKYGQK or WRKYGKK) and the C_2_HC domain (CX_7_CX_24_HX_1_C) or C_2_H_2_ domain (CX_5_CX_23_HX_1_H) are highlighted by the blue and purple rectangles, respectively.

The cDNA sequence of the *ScWRKY5* gene (GenBank Accession No. MK629767) with a total length of 1359 bp was successfully obtained from the mature leaves of the sugarcane variety ROC22. The ScWRKY5 protein encodes 238 amino acid residues and has a theoretical molecular weight of 26.78 kDa. The isoelectric point, average hydrophobicity, and instability coefficient of the ScWRKY5 protein were 6.11, − 0.611, and 56.46, respectively, suggesting that ScWRKY5 is an acidic, unstable, and hydrophilic protein. Amino acid sequence alignment indicated that the ScWRKY5 protein has low homology with previously reported class II sugarcane WRKYs (Sc-WRKY, ScWRKY3, ScWRKY4, and ScWRKY6), which was 7.66%, 8.98%, 10.08%, and 8.76%, respectively (Fig. 1A). The amino-acid sequence similarity of the ScWRKY5 protein to *S. bicolor* SbWRKY32 (XP_002456546.1), *Z. mays* ZmWRKY22 (NP_001147816.1), *S. italica* SiWRKY29 (XP_004972233.1), *O. sativa* OsWRKY64 (XP_015642042.1), *Triticum urartu* TuWRKY38 (EMS45972.1), and *Brachypodium distachyon* BdWRKY38 (XP_010233534.1) was 94.09%, 85.12%, 81.30%, 61.54%, 57.53%, and 54.66%, respectively (Fig. 1B). A conserved WRKY domain (WRKYGQK) and a zinc-finger-like motif (CX_7_CX_24_HX_1_C) were found at the C-terminus of these seven WRKY proteins (Fig. 1B), whereas a WRKY domain (WRKYGKK or WRKYGQK) and a zinc-finger-like motif (CX_7_CX_24_HX_1_C or CX_5_CX_23_HX_1_H) were contained at the four reported class II sugarcane WRKYs (Fig. 1A). Phylogenetic tree analysis showed that ScWRKY5 and the WRKY family proteins from *S. spontaneum* could be divided into three categories (I–III) (Fig. S1). The ScWRKY5 protein was classified into group III and showed 93.99% and 93.70% amino acid sequence similarity with the *S. spontaneum* WRKY (Sspon.03g0003780-2c) and *S. bicolor* SbWRKY35 (Sb03g038170) (Figs. S1 and 2). The *cis*-acting elements contained in the promoter of *S. spontaneum* WRKY (Sspon.03g0003780-2c) were predicted online using the PlantCARE tool. The upstream 2000 bp promoter of the *S. spontaneum WRKY* (Sspon.03g0003780-2c) contained 20 types of *cis*-acting elements (Table S1), including a large number of the core promoter elements TATA-box (54 sites) and CAAT-box (21 sites), as well as many other types of *cis*-acting elements. There were eight types of light-response elements, which were the *cis*-acting elements of the GA-motif, GATA-motif, 4 cl-CMA2b, GT1-motif, G-Box, G-box, box 4, and AE-box. Several response hormone signal substances were predicted, such as the TGACG-motif, P-box, ABRE, and TCA-element, which may participate in MeJA, gibberellin (GA), SA, and ABA reactions, respectively. There were also some elements related to the abiotic stress response, such as MBS *cis*-acting elements involved in drought stress. The promoter element and functional prediction indicated that *ScWRKY5*, the homologous gene of the *S. spontaneum WRKY* (Sspon.03g0003780-2c), may be regulated by photosystem, hormone signaling substances, and abiotic stresses.

**Figure 2:**
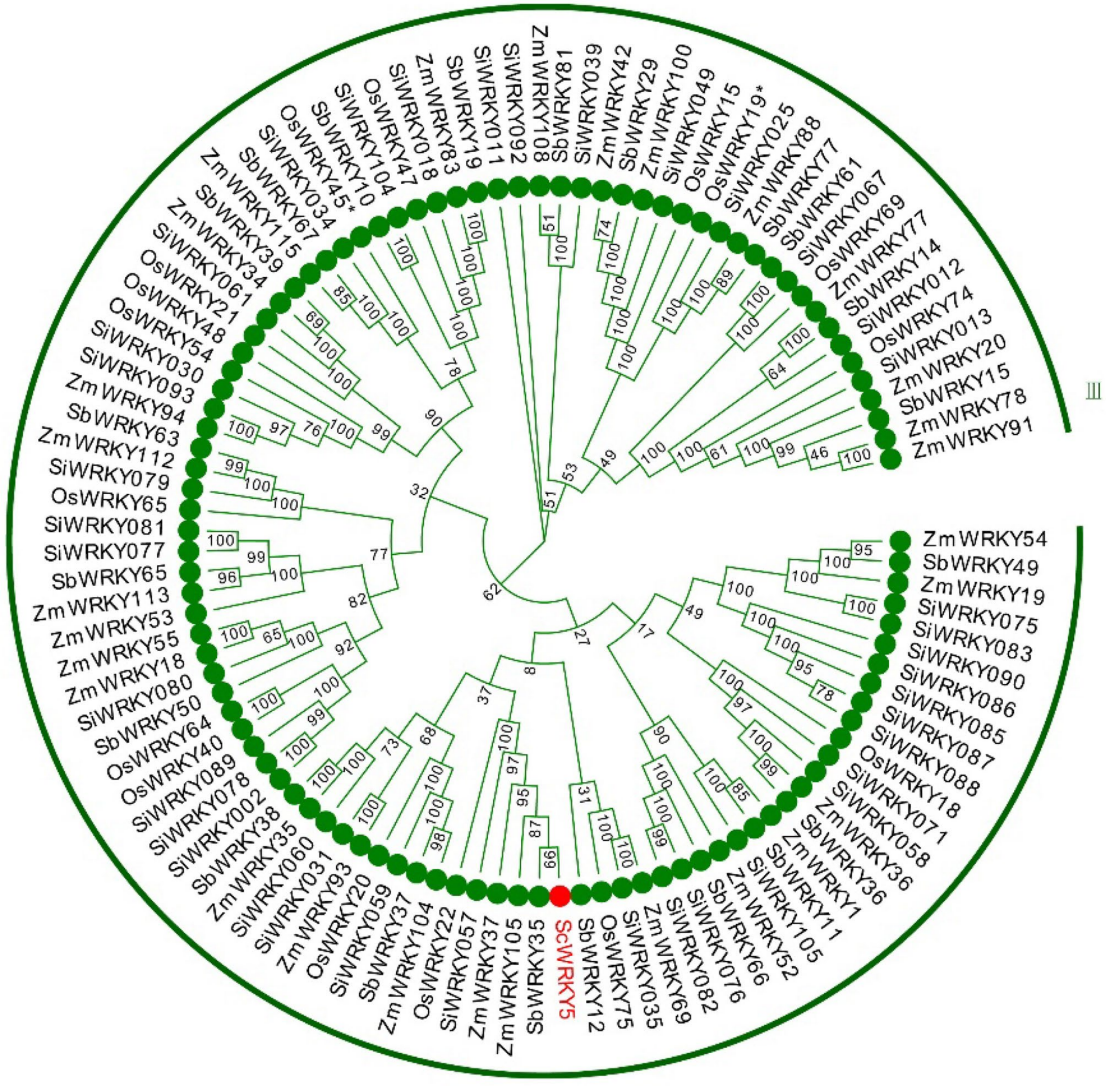
Phylogenetic tree analysis of the ScWRKY5 protein and WRKYs from other plant species. The phylogenetic tree was plotted using the neighbor-joining (NJ) method with 1000 bootstrap replicates in MEGA 7.0 software. The ScWRKY5 protein is marked by a red circle. ZmWRKY, SbWRKY, SiWRKY, and OsWRKY represent the WRKYs from *Zea mays*, *Sorghum bicolor*, *Setaria italica*, and *Oryza sativa*, respectively.

### Subcellular localization of ScWRKY5

**Figure 3:**
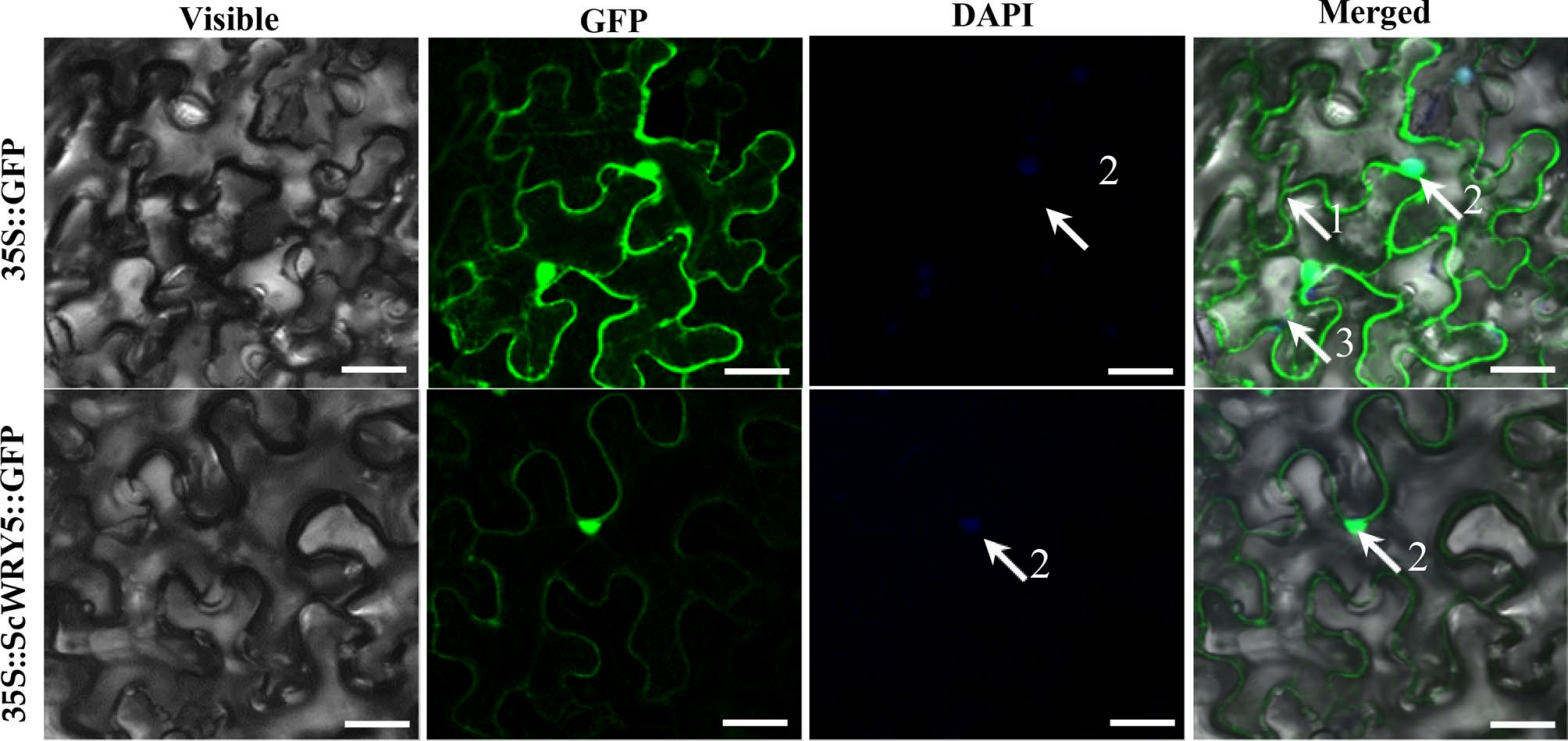
Subcellular localization of ScWRKY5 in *Nicotiana benthamiana* leaves. The epidermal cells of *N. benthamiana* were used for capturing images of visible light, green fluorescence, blue fluorescence, and visible light merged with green and blue fluorescence. White arrows 1, 2, and 3 indicate the cell membrane, nucleus, and cytoplasm, respectively. Scale bar = 50 μm. *35S::GFP*, the *Agrobacterium tumefaciens* strain carrying the empty vector pMDC83-*GFP. 35S::ScWRKY5::GFP*, the *A. tumefaciens* strain carrying the recombinant vector pMDC83-*ScWRKY5*-*GFP*. GFP, green fluorescent protein; DAPI, 4′,6-diamidino-2-phenylindole.

The Euk-mPLoc 2.0 Server predicted the subcellular localization of the sugarcane ScWRKY5 protein in the nucleus. However, the WoLF PSORT predicted the subcellular localization of the ScWRKY5 protein in the nucleus with the highest probability, followed by in the chloroplast, cytoplasm, and peroxisome. Here, to validate the true location of the ScWRKY5 protein, the fusion expression plasmid pMDC83-*ScWRKY5*-*GFP* was constructed, and the subcellular localization expression vector pMDC83-*GFP* was used as a positive control. Using *Agrobacterium*-mediated transformation and injecting into *N. benthamiana* leaves, the ScWRKY5-GFP fusion protein emitted green fluorescence predominantly in the nucleus, whereas the fluorescence of free GFP was detected in almost around all cell space (Fig. 3). These results indicated that the ScWRKY5 protein is localized in the nucleus.

### Transcriptional activation activity of ScWRKY5

**Figure 4:**
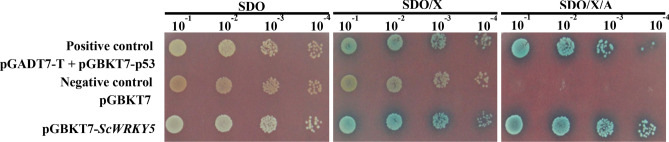
The ScWRKY5 transactivation activity test. SDO (SD/-Trp), synthetic dropout medium without tryptophan; SDO/X (SD/-Trp/X-α-Gal), synthetic dropout medium without tryptophan, but plus 5-bromo-4-chloro-3-indoxyl-α-D-galactopyranoside; SDO/X/A (SD/-Trp/X-α-Gal/AbA), synthetic dropout medium without tryptophan but plus 5-bromo-4-chloro-3-indoxyl-α-D-galactopyranoside and aureobasidin A. pGADT7-T + pGBKT7-p53, positive control; pGBKT7, negative control.

The Y2HGold-GAL4 yeast two-hybrid system was used to analyze the self-activating activity of the target protein. On the SDO (synthetic dropout medium without tryptophan) plate, the positive control pGADT7 + pGBKT7-p53 and the negative controls pGBKT7 and pGBKT7-*ScWRKY5* could grow normally, indicating that the target plasmid pGBKT7-*ScWRKY5* had been successfully transferred into the yeast Y2HGold, and tryptophan could be successfully expressed in GAL4-BD combined with the ScWRKY5 protein (Figs. 4 and S2). On the SDO plate with X-α-Gal dye added, the positive control and the yeast cells transformed with pGBKT7-*ScWRKY5* all turned blue, suggesting that the *MEL1* gene had been activated. Further tests showed that the positive control grew normally and turned blue on the SDO/X plate medium with the addition of the aureobasidin A (AbA), indicating that the reporter genes *AUR1-C* and *MEL1* had been activated. The blue colonies of the yeast cells transformed with pGBKT7-*ScWRKY5* proved that the protein of ScWRKY5 exhibited transcriptional activation activity.

### Tissue-specific expression of *ScWRKY5* in sugarcane

**Figure 5:**
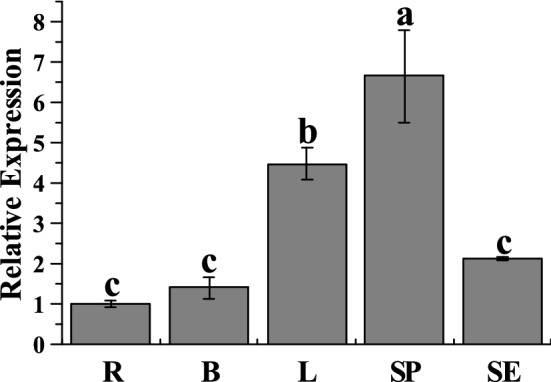
Tissue-specific expression of *ScWRKY5* in different sugarcane tissues. The root, bud, leaf, stem pith, and stem epidermis tissues are represented by R, B, L, SP, and SE, respectively.

The relative expression level of the *ScWRKY5* gene in the sugarcane root, bud, leaf, stem pith, and stem epidermis was detected by quantitative real-time PCR (qRT-PCR) (Fig. 5). The results indicated that this gene was constitutively expressed in all of these tissues. The lowest expression level of *ScWRKY5* occurred in the root, while its relative expression level in the stem pith and leaf was 6.61-fold and 4.45-fold higher than that in the root, respectively.

### Expression patterns of *ScWRKY5* under different stresses

**Figure 6:**
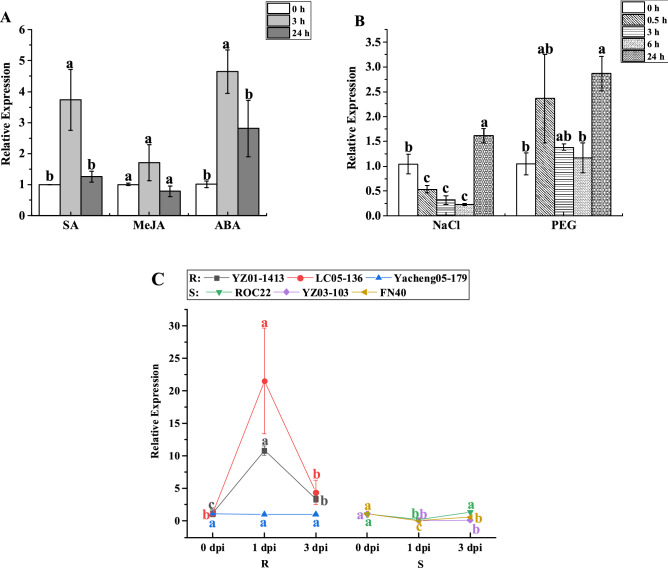
Expression patterns of *ScWRKY5* under different stresses as determined by qRT-PCR. (**A**) Relative expression of *ScWRKY5* in 4-month-old ROC22 plantlets under plant hormone stress. SA, salicylic acid (5 mM); MeJA, methyl jasmonate (25 µM); ABA, abscisic acid (100 µM). (**B**) Relative expression of *ScWRKY5* in 4-month-old ROC22 plantlets under abiotic stress. NaCl, sodium chloride (simulating salt stress) (250 mM); PEG, polyethylene glycol (simulating drought treatment) (25%). (**C**) Relative expression of *ScWRKY5* in sugarcane after inoculation with *Sporisorium scitamineum*. YZ01-1413, LC05-136, and Yacheng05-179 were smut-resistant sugarcane varieties (R). ROC22, YZ03-103, and FN40 were smut-susceptible sugarcane varieties (S). dpi, days post-inoculation. Data are normalized to the glyceraldehyde-3-phosphate dehydrogenase (*GAPDH*) expression level. All of the data points are means ± standard error (*n* = *3*). Bars superscripted by different lowercase letters indicate significant differences, as determined by Duncan’s new multiple range test (*p*-value < 0.05).

The qRT-PCR method was used to analyze the expression patterns of the *ScWRKY5* gene under different exogenous stresses (Fig. 6). Under 5 mM SA stress for 3 h, the gene expression level of *ScWRKY5* was 3.74-fold higher than that of the control (Fig. 6A). Within 24 h of 100 μM MeJA treatment, the gene expression level of *ScWRKY5* was relatively stable (Fig. 6A). Under 100 μM ABA stress, the gene expression level of *ScWRKY5* was significantly increased at 3 h, being 4.65-fold higher than that of the control, following which it dropped to the control level at 24 h (Fig. 6A). Under 250 mM NaCl stress, the gene expression level of *ScWRKY5* was stabilized from 0.5 h to 6 h and then rose rapidly to a peak at 24 h, at which point it was 1.62-fold higher than that of the control (Fig. 6B). Under 25% PEG 8000 stress, the transcription of *ScWRKY5* remained unchanged at 0.5–6 h, but was increased to a maximum at 24 h which was 1.56-fold higher than that of the control (Fig. 6B). In addition, the transcripts of *ScWRKY5* was detected in the interaction between six different sugarcane varieties and *S. scitamineum* (Fig. 6C). In the smut-resistant varieties, the expression level of *ScWRKY5* was significantly increased in YZ01-1413 and LC05-136 after inoculation with the *S. scitamineum* for 1 day post-inoculation (dpi), which were 8.58- and 20.32-fold higher than that in the control group, respectively. However, the expression level of *ScWRKY5* did not change significantly within 3 dpi in the smut-resistant variety Yacheng05-179. Among the three smut-susceptible varieties, the expression level of *ScWRKY5* was significantly decreased in YZ03-103 and FN40 from 1 to 3 dpi, while it was significantly decreased in ROC22 at 1 dpi and returned to the control level at 3 dpi. These results revealed that the transcripts of *ScWRKY5* could be induced by ABA, NaCl, PEG, SA, and *S. scitamineum* stresses.

### Transient overexpression of *ScWRKY5* in *N. benthamiana* leaves

**Figure 7:**
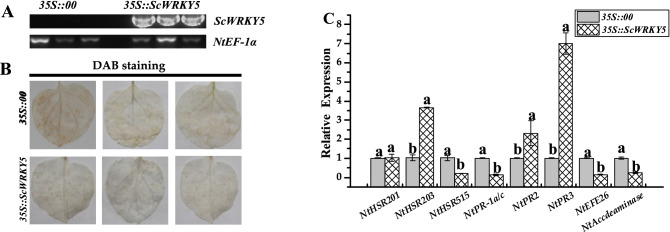
Transient overexpression of *ScWRKY5* in *Nicotiana benthamiana* leaves. (**A**) Semi-quantitative PCR analysis of *ScWRKY5* in *N. benthamiana* leaves inoculated with *Agrobacterium* GV3101 carrying pEarleyGate 203-*ScWRKY5* (*35S::ScWRKY5*) and the empty vector pEarleyGate 203 (*35S::00*). (**B**) DAB staining analysis of *N. benthamiana* leaves after one day of inoculation. (**C**) Transcription levels of eight immune-related marker genes in *N. benthamiana* leaves after one day of inoculation. Data are normalized to the expression level of *NtEF-1α*. All data points are means ± standard error (*n* = 3). Bars superscripted by different lowercase letters indicate significant differences, as determined by Duncan’s new multiple range test (*p*-value < 0.05).

The transient overexpression of *A. tumefaciens* cells carrying *ScWRKY5* in *N. benthamiana* leaves was performed. The transcript of the *ScWRKY5* gene was successfully detected in the *N. benthamiana* leaves using a semi-quantitative PCR technology (Figs. 7A and S3). The DAB (3,3′-diaminobenzidine) test showed that the staining color in the *35S::ScWRKY5* leaves was nearly the same as that of the control (Fig. 7B). The qRT-PCR results (Fig. 7C) indicated that in the *35S::ScWRKY5* leaves, the expression levels of the hypersensitive response (HR) marker gene *NtHSR203*, and JA pathway related genes *NtPR2* and *NtPR3* were all increased, which were 3.56-, 2.30-, and 7.00-fold higher than that of the control group, respectively. But the HR marker gene *NtHSR515*, the SA pathway related gene *NtPR-1a/c*, and the ET synthesis-dependent genes *NtEFE26* and *NtAccdeaminase* were all decreased, which were 0.20-, 0.13-, 0.14-, and 0.22-fold lower than that of the control group, respectively. In addition, the expression level of HR marker gene *NtHSR201* was relatively stable.

**Figure 8:**
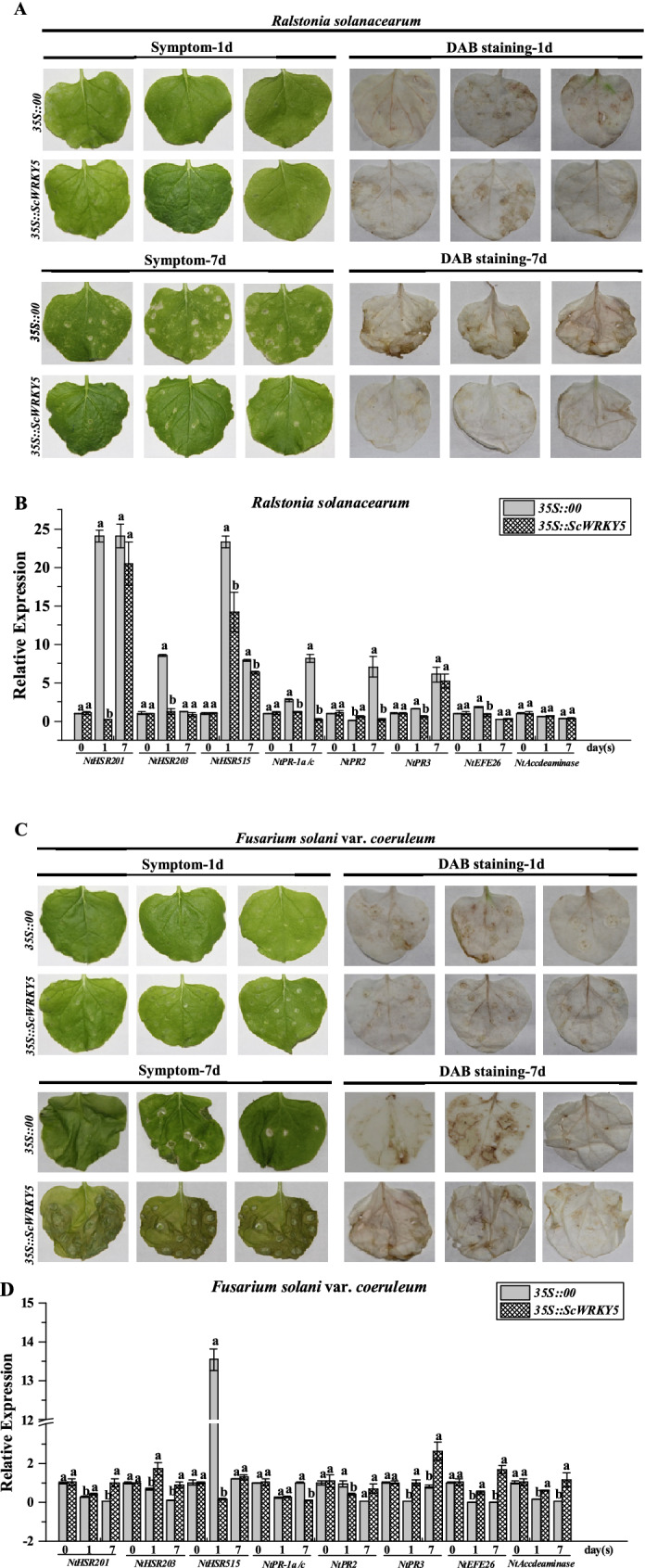
Resistance effect of the transient overexpression of *ScWRKY5* in *Nicotiana benthamiana* leaves. (**A**,**C**) Phenotype and DAB staining analysis of *N. benthamiana* leaves inoculated with *Ralstonia solanacearum* and *Fusarium solani* var. *coeruleum* after one day and seven days of inoculation. (**B**,**D**) Transcription levels of eight immune-related marker genes in *N. benthamiana* leaves after one day and seven days of inoculation with *R. solanacearum* and *F. solani* var. *coeruleum*. Data are normalized to the expression level of *NtEF-1α*. All of the data points are means ± standard error (*n* = 3). Bars superscripted by different lowercase letters indicate significant differences, as determined by Duncan’s new multiple range test (*p*-value < 0.05).

Phenotype analysis showed that there was no obvious symptom in the *35S::00* and *35S::ScWRKY5* leaves at one-day after inoculation with *R. solanacearum*. However, the leaves were yellow and the allergic spots were more serious in the *35S::00* group after seven days (Fig. 8A). The qRT-PCR results indicated that the expression level of the *NtPR2* gene was increased by the overexpression of the *ScWRKY5* gene at one-day post inoculation, which was 124.46-fold higher than that of the control group, and was repressed after seven days. The expression levels of the *NtHSR201*, *NtHSR203*, *NtHSR515*, *NtPR-1a/c*, *NtPR3*, and *NtEF26* were repressed at one-day post inoculation, and recovered to the control level after seven days. The expression level of the *NtAccdeaminase* remained unchanged at one and seven days post inoculation (Fig. 8B).

The *N. benthamiana* leaves overexpressing the *ScWRKY5* gene did not show symptoms after one day of inoculation with *F. solani* var. *coeruleum*. Conversely, the leaves turned yellow and wilted severely after seven days of inoculation, which was higher than that of the control group (Fig. 8C). The DAB results showed that as the infection time increased, the brown color of the tobacco darkened, and the leaves overexpressing the *ScWRKY5* gene were darker than the control group after seven days. (Fig. 8C). The qRT-PCR showed that, compared with the control group, the expression level of *NtHSR515* and *NtPR2* was decreased in the *35S::ScWRKY5* leaves after inoculation for one day, and the expression level of *NtPR-1a/c* was stable at the control level. Instead, the expression levels of *NtHSR201*, *NtHSR203*, *NtPR3*, *NtEFE26*, and *NtAccdeaminase* were significantly increased in the *35S::ScWRKY5* leaves, which were 1.55-, 2.54-, 22.17-, 1381.62-, and 3.55-fold higher than that of the control group, respectively. After seven days of infection, only the expression level of *NtPR-1a/c* was decreased, while the expression of *NtHSR515* and *NtPR2* was stable at the control level, and the expression of the other five genes was still increased (Fig. 8D).

## Discussion

WRKY is one of the largest transcription factors in plants and plays an important role in the plant response to biotic and abiotic stresses. As more WRKY transcription factors are being identified from different plants, their structural characteristics, origin, evolution, and gene functions are being increasingly studied^3,18^. However, there are only limited studies on *WRKY* genes in sugarcane^30–34^. In this study, the *ScWRKY5* gene was isolated from sugarcane ROC22. The ScWRKY5 protein was clustered with the amino acid sequences from *S. spontaneum* in the class III WRKY family^34^ (Fig. S1). The physical and chemical properties of proteins are of great significance to their molecular biological functions. We predicted that the sugarcane ScWRKY5 was a hydrophilic protein with poor functional stability. Although ScWRKY5 belongs to the class III WRKY family, it only has one zinc-finger-like motif CX_7_CX_24_HX_1_C, which is the same as that of tomato SlWRKY80^35^. As reported, only one of 81 tomato WRKY proteins has this zinc-finger-like motif (CX_7_CX_24_HX_1_C) variation^35^, suggesting that this type of structural variation in zinc finger is uncommon. Therefore, it is necessary to further assess whether the variant zinc structure of ScWRKY5 will cause the appearance or loss of some specific functions.

The location of proteins in cells is important for determining their biological functions^36^. The subcellular localization analysis of 164 NtWRKY transcription factors in common tobacco showed that 118 NtWRKYs (including most of the class I and class II WRKY family members) were located in the nucleus, and 77.4% of class III WRKYs were located in the cytoplasm^37^. In this study, the ScWRKY5-GFP fusion protein emitted green fluorescence predominantly in the nucleus (Fig. 3) which is consistent with the result predicted by the Euk-mPLoc 2.0 Server, suggesting that the ScWRKY5 protein may play the role of a nucleoprotein in sugarcane. However, for the small probability that ScWRKY5 protein was predicted to locate in the chloroplast, cytoplasm, or peroxisome by WoLF PSORT software, further subcellular localization test is needed in conjunction with promoter analysis of the *ScWRKY5* gene itself as well as a western blot to check the integrity of the fusion protein in the tissues infiltrated.

Transcriptional activity is important for the functional analysis of transcription factors^38^. Since the GAL4 yeast two-hybrid system was firstly discovered^39^, this method has been increasingly used for studying the interactions between WRKY proteins^40^. In the present study, the protein encoded by the *ScWRKY5* cDNA showed a self-activating activity (Fig. 4). Similarly, GhWRKY40 not only has a self-activating activity in yeast, but it also acts as a downstream interaction protein of GhMPK20, participating in plant-borne pathogenic signals through the MAPK cascade pathway^41^. Proteins with self-activation activity can be detected in sections to determine the specific location of the self-activation region of the protein, which can be verified in subsequent experiments.

The expression abundance of the *WRKY* gene in different plant tissues is not consistent^42–44^. There are 28 *CiWRKY* genes detected in the roots, stems and leaves of *Caragana intermedia*^42^. Different *CiWRKY* genes are expressed in different tissues, e.g., *CiWRKY69-1* had the highest expression level in the roots, while *CiWRKY40-1* and *CiWRKY30* were mainly expressed in the leaves^42^. Bakshi et al.^20^ found that 12 of the 37 *Arabidopsis WRKY* genes were specifically expressed in the mature region of the root cells and may be involved in regulating the maturation of *Arabidopsis* root cells. *TaWRKY44* was expressed differently in all of the tested organs, including the roots, stems, leaves, pistils, and stamens, with the highest expression level in the leaves and the lowest in the pistil^43^. In this study, *ScWRKY5* was expressed constitutively in the sugarcane tissues, with the highest expression level in the stem pith, and the lowest level in the root (Fig. 5). These results indicated that *WRKY* genes may play different roles in the regulation of plant growth and development.

WRKY transcription factors can rapidly perceive signals of changes in external environmental conditions and transmit these signals in a timely manner in order for plants to respond to the environment^12,45^. Abiotic stresses, such as salt and drought can induce the expression of a series of stress response genes in the ABA signal transduction pathway, thereby enhancing the plant resistance^46^. Wang et al.^31^ found that the expression level of the *ScWRKY3* gene was increased under the stresses of NaCl, PEG, and the plant hormone ABA, but it was repressed under the stresses of SA and MeJA. In this study, the expression level of the *ScWRKY5* gene was also increased by the stresses of PEG, NaCl, SA, and ABA (Fig. 6). It is speculated that the *ScWRKY5* gene may play a role in the tolerance to salt and drought in sugarcane through SA and ABA signaling pathways. Similarly, under drought and salt conditions, the expression level of *SlWRKY8* in *Solanum lycopersicum* is significantly increased, which occurs by directly activating the expression of *SlWRKY8* in the SA and ABA synthesis pathways, thereby enhancing the resistance of *S. lycopersicum* to drought and salt stresses^47^.

Previous studies have shown that the *GhWRKY40* gene is induced by the bacteria *R. solanacearum* upon infection and is up-regulated after treatment with SA, MeJA, and ET^41^. When *GhWRKY40* was transiently overexpressed in tobacco leaves, most resistance-related genes, including SA, ET, JA, and HR response genes, were repressed after infection with *R. solanacearum*, suggesting that the overexpression of *GhWRKY40* reduce the resistance to *R. solanacearum*^41^. Overexpressing *AtWRKY28* and *AtWRKY75* increased the tolerance to oxalic acid and the resistance to *Sclerotinia sclerotiorum* by participating in JA/ET-dependent defense signaling pathways in *Arabidopsis*^48^. The *OsWRKY13* gene enhanced the defense ability of rice blast via cross-talk with the SA-dependent defense pathway, *SNAC1*-mediated abiotic stress defense pathway, and JA signaling pathway^49^. When infected with *R. solanacearum*, *Arabidopsis WRKY27* has a negative regulatory effect on the expression of defense-related genes and exhibited earlier disease symptoms than the mutant *WRKY27-1* plant, which had lost *WRKY27*^50^. Overexpressing *GhWRKY44* induced the expression of several defense-related genes in transgenic plants, including *PR-1*, *PR-2*, *PR-5*, and *NPR1* in the SA pathway and *PR-4* in the JA pathway, and enhanced the resistance of transgenic tobacco to *R. solanacearum* and *Rhizoctonia solani*^51^. After infected with *Phytophthora nicotianae*, the overexpression of *SpWRKY1* enhanced the expression level of SA- and JA-associated genes (*NtPR1*, *NtPR2*, *NtPR4*, *NtPR5*, and *NtPDF1.2*), suggesting that *SpWRKY1* acts as a positive regulator in tobacco defense responses to *P. nicotianae*^52^. The overexpression of *NtPR-Q* in tobacco enhanced the expression of SA dependent genes (*NtPR1a/c*, *NtPR2*, and *NtCHN50*), thus enhanced the resistance of plants to *R. solanacearum*^53^. In transgenic tobacco plants, the symptom of leaf wilting was lighter than that of wild type plants, and the expression levels of HR-associated gene *HSR515* and SA-dependent marker genes *NPR1* and *PR2* were continuously upregulated by the ectopic overexpression of *CaC3H14*, indicating that *CaC3H14* played a positive regulatory role in resistance to *R. solanacearum*^54^. In this study, the expression level of the *NtPR2* gene was increased in the *35S::ScWRKY5* leaves at one-day post inoculation with *R. solanacearum* (Fig. 8B). Compared with the *35S::ScWRKY5*, the leaves were yellow and the allergic spots were more serious in the control group after seven days of inoculation with *R. solanacearum* (Fig. 8A). These suggested that the transient overexpression of *ScWRKY5* may enhance the resistance of *N. benthamiana* to *R. solanacearum* by participating in SA-dependent defense signaling pathways. After inoculation with *F. solani* var. *coeruleum*, the wilting disease symptoms were greater in *35S::ScWRKY5* leaves than in the control. Furthermore, the expression levels of the HR marker gene *NtHSR515*, and the SA pathway related genes *NtPR-1a/c* and *NtPR2* were decreased in *35S::ScWRKY5* leaves (Fig. 8D). The results revealed that the transient overexpression of *ScWRKY5* may reduce the resistance of *N. benthamiana* to *F. solani* var. *coeruleum* via repressed the expression of HR and SA pathway related genes. The regulation mechanism of the *ScWRKY5* gene in disease resistance need to be further verified after stable genetic transformation. In summary, the *ScWRKY5* gene may be involved in plant ABA, SA, and ET signal regulatory networks and plays a role in the plant response to biotic and abiotic stresses. However, more *WRKY* family genes in sugarcane need to be discovered and identified, which is more conducive to the further study of the possible functional differentiation between different family members of *WRKY* genes.

## Methods

### Plant materials and methods

In order to analyze the tissue-specific expression level of *ScWRKY5*, nine 10-month-old ROC22 (the main sugarcane variety in mainland China) plants were randomly selected from the field based on the method of Huang et al.^55^. The sugarcane white root, bud, stem pith, stem epidermis, and + 1 leaf were collected and frozen in liquid nitrogen, and then stored at − 80 °C until use. Each sample contained three biological replicates, and each biological replicate contained three plants.

To detect the expression level of *ScWRKY5* in sugarcane inoculated with smut pathogen, six different sugarcane varieties, including three smut-resistant varieties (YZ01-1413, LC05-136, and Yacheng05-179) and three smut-susceptible varieties (ROC22, YZ03-103, and FN40), were provided by the Key Laboratory of Sugarcane Biology and Genetics and Breeding, Ministry of Agriculture, Fujian Agriculture and Forestry University^56^. The 10-month-old sugarcane stalks were selected and cultured at 32 °C. After the buds had germinated to 1–2 cm in length, an acupuncture inoculation method with 5 × 10^6^ mL^−1^ (containing 0.01% Tween-20, *v/v*) smut pathogen suspension was used to inoculate the sugarcane buds, and the control group was inoculated with sterile water (containing 0.01% Tween-20, *v/v*)^57^. All of the samples were cultured at 28 °C for 16 h in the light and 8 h in the dark. Five single buds were collected at 0, 1, and 3 dpi, frozen in liquid nitrogen, and stored at − 80 °C for future use.

For the analysis of the expression patterns of *ScWRKY5* under different exogenous stresses, four-month-old ROC22 tissue culture seedlings were cultivated in water for about 10 d. Two groups of the seedlings were cultured in aqueous solutions of 250 mM NaCl and 25% PEG 8000 solutions, and the leaves were sampled at 0, 0.5, 3, 6, and 24 h, respectively^57–59^. The leaves of the other three groups of the seedlings were sprayed with 5 mM SA (with 0.01% Tween-20, *v/v*), 25 μM MeJA (containing 0.1% ethanol and 0.05% Tween-20, *v/v*), and 100 μM ABA, respectively^57–59^. Under the SA, MeJA and ABA treatments, the leaves were collected at 0, 3, and 24 h. All of the samples were frozen in liquid nitrogen and stored at − 80 °C until use. Each sample contained three biological replicates, and each biological replicate contained three seedlings.

### Total RNA extraction and cDNA first-strand synthesis

Total RNA of the sugarcane samples was extracted using the TRIzol method^60^. RQ1 RNase-Free DNase reagent was used to remove the DNA from the total RNA of the sample. The cDNA of the mature leaves of sugarcane ROC22, a template for *ScWRKY5* gene cloning, was reverse-transcribed according to the RevertAid First Strand cDNA Synthesis Kit (Fermentas, Shanghai, China). Referring to the instruction manual of the Prime-Script RT Reagent Kit (TaKaRa Biotechnology, Dalian, China), the first-strand cDNA of the sugarcane tissues and different materials under various stresses was reversed and used to quantitatively detect the expression level of the target gene. The obtained RNA and cDNA were tested by 1.0% agarose gel electrophoresis.

### Cloning, sequencing and bioinformatics analysis of *ScWRKY5*

From our previous transcriptome data of sugarcane^61^, a gene encoding a predictable WRKY transcription factor *ScWRKY5* was screened. The National Center of Biotechnology Information (NCBI) online software (https://www.ncbi.nlm.nih.gov/tools/primer-blast/) was used to design specific cloning amplification primers (Table S2). The cDNA of the mature leaves of ROC22 was used as a template, and a PCR reaction system was prepared according to the *ExTaq* DNA reagent instructions. The PCR program was set as 94 °C pre-denaturation for 4 min; followed by 35 cycles of 94 °C denaturation for 30 s, 59.5 °C (decreasing by 0.1 °C in each cycle) for 30 s, a 72 °C extension for 2 min; and then a final extension at 72 °C for another 10 min. The PCR reaction product was detected by agarose gel electrophoresis, purified, and ligated to the cloning vector pMD19-T (TaKaRa Biotechnology, Dalian, China). The correct recombinant plasmid after sequencing (Fuzhou Shangya Biotechnology Co., Ltd.) was named pMD19-T-*ScWRKY5*. ORF Finder (https://www.ncbi.nlm.nih.gov/orffinder/) and conserved domains database (CDD) (http://www.ncbi.nlm.nih.gov/Structure/cdd/wrpsb.cgi) ^62^ were used to analyze the ORF of the *ScWRKY5* gene and the conserved domain. The BLASTp program (https://blast.ncbi.nlm.nih.gov/Blast.cgi?PROGRAM=blastp&PAGE_TYPE=BlastSearch&LINK_LOC=blasthome) was used to find homologous amino acid sequences from other plant species. DNAMAN 6.0.3.99 software was used to perform sequence homology analysis. ProtParam (https://web.expasy.org/protparam/) ^63^ and NPS@ server (https://npsa-prabi.ibcp.fr/cgi-bin/npsa_automat.pl?page=/NPSA/npsa_hnn.html) ^64^ were used to predict the primary structure and secondary structure of the target protein, respectively. Euk-mPLoc 2.0 Server (http://www.csbio.sjtu.edu.cn/bioinf/euk-multi-2/) ^65^ and WoLF PSORT (https://wolfpsort.hgc.jp/) ^66^ were both used for predicting the subcellular localization of the target protein. The neighbor-joining (NJ) (1000 bootstrap replicates) method in MEGA 7.0 software^67^ was used to construct phylogenetic trees of ScWRKY5 protein and other WRKY proteins from *S. bicolor*^68^, *Z. mays*^69^, *S. italica*^18^, *O. sativa*^70^, and *S. spontaneum*^34^. Nucleotide BLAST (https://blast.ncbi.nlm.nih.gov/Blast.cgi?PROGRAM=blastn&PAGE_TYPE=BlastSearch&LINK_LOC=blasthome) was used to extract the promoter sequence of the *S. spontaneum WRKY* gene (Sspon.03g0003780-2c). The Plant CARE (http://bioinformatics.psb.ugent.be/webtools/plantcare/html/) ^71^ online software was used to predict the promoter element and function of the *S. spontaneum WRKY* gene (Sspon.03g0003780-2c).

### Subcellular localization analysis

The Gateway entry vector primer pair ScWRKY5-Gate-F/R (Table S2) was designed based on the sequences of the *ScWRKY5* ORF (without the stop codon) and the entry vector pDONR-221. After PCR amplification, the gel-purified product was subjected to the entry vector pDONR-221 by BP reaction to obtain a positive plasmid pDONR-221-*ScWRKY5*. Subsequently, after *Ssp* I single digestion and purification, *ScWRKY5* was subjected to the subcellular localization vector pMDC83^72^ by LR reaction. After sequencing, the recombinant subcellular localization vector *35S::ScWRKY5::GFP* was obtained.

The positive recombinant plasmid *35S::ScWRKY5::GFP* was transformed into *A. tumefaciens* GV3101 strain. The *Agrobacterium* cells were collected and diluted to OD_600_ = 0.8 with MS blank medium. A 1.0-mL sterile syringe was used to transfer the bacterial solution (containing 200 μM acetylsyringone) into the 5–8 leaves of *N. benthamiana*^73,74^. After culturing at 28 °C for 16 h in the light and 8 h in the dark for 2 d, the injected leaves were removed, and 1.0 μM 4′, 6-diamidino-2-phenylindole (DAPI) was used for nuclear staining. A Leica TCS SP8 laser confocal microscope [Leica Microsystems (Shanghai) Trading Co., Ltd., Mannheim, Germany] with a 10 × lens, 488 nm green fluorescence excitation wavelength, and 458 nm chromatin DAPI filter was used to observe the subcellular localization^75,76^.

### Analysis of ScWRKY5 transcriptional self-activating activity

The target gene *ScWRKY5* was constructed on the pGBKT7 vector, and the recombinant plasmid pGBKT7-*ScWRKY5* was digested with enzymes and verified by sequencing analysis. A primer pair *ScWRKY5*-BD-F/R (Table S2) containing a specific restriction site was designed based on the sequences of *ScWRKY5* ORF (without the stop codon) and the DNA-binding domain (BD) vector pGBKT7. The pMD19-T-*ScWRKY5* plasmid was used as a template for PCR amplification. The gel-purified product and the pGBKT7 vector plasmid were digested with the *Nde* I and *Eco*R I enzymes, (Table S2) and then purified. Subsequently, the *ScWRKY5* gene was ligated to the vector pGBKT7 by using T4 DNase, and transformed into DH5α competent cells. After bacterial liquid PCR detection, enzyme digestion verification, and sequencing comparison, the pGBKT7-*ScWRKY5* recombinant plasmid was obtained.

The Y2HGold-GAL4 yeast two-hybrid system (containing four reporter genes *AUR1-C*, *HIS3*, *ADE2*, and *MEL1*) was used to analyze the transcriptional self-activating activity of ScWRKY5. According to the instructions of the Y2HGold Chemically Competent Cell, the positive hybridization vector plasmid pGBKT7-53 + pGADT7-T, the negative plasmid empty pGBKT7, and the constructed target fusion expression plasmid pGBKT7-*ScWRKY5* were used to transform yeast Y2HGold competent cells in SDO (SD/-Trp, tryptophan-deficient medium) and cultured on a plate at 29 °C for two days, until the bacteria grew. Then the bacteria solutions were diluted by 10^–1^, 10^–2^, and 10^–3^, and 8.0 µL each of them were spotted on SDO (SD/-Trp, tryptophan-deficient medium), SDO/X (SD/-Trp/X-α-Gal, tryptophan-deficient medium supplemented with 5-bromo-4-chloro-3-indole-α-D-galactoside), and SDO/X/A (SD/-Trp/X-α-Gal/AbA, tryptophan-deficient medium supplemented with 5-bromo-4-chloro-3-indole-α-D-galactoside and astratin A) plates at 29 °C. The growing colonies were photographed, and the self-activation of the target protein was analyzed according to the growth status and color changes of the colonies.

### Expression patterns of *ScWRKY5* in sugarcane tissues and under various stresses

The 15-fold diluent cDNA of different sugarcane tissues (root, bud, leaf, stem pith, and stem epidermis) and samples treated with ABA, SA, MeJA, PEG, NaCl, and *S. scitamineum* was used as the qRT-PCR template. Based on the *ScWRKY5* gene sequence, the specific qRT-PCR primer pair ScWRKY5-QF/R (Table S2) was designed on the NCBI online software. Glyceraldehyde-3-phosphate dehydrogenase (*GAPDH*, GenBank Accession No. CA254672) was used as the internal reference gene (Table S2). An ABI 7500 Real-Time PCR System (Applied Biosystems, Foster City, CA, USA) was used for qRT-PCR detection, and a quantitative reaction system was prepared according to the SYBR Green PCR Master Mix Kit instructions (Roche, Shanghai, China). Three technical replicates were set up for each sample, and sterile water was used as a template in the negative control. The relative expression of the target gene was calculated using 2^-△△Ct^ method^76^. The significance level of the data was analyzed using DPS 9.50 software, and graphs were plotted using Origin 8.0 software. In the smut pathogen infection test, the relative expression of the target gene was calculated with reference to the method of Su et al.^77^.

### Transient expression of *ScWRKY5* in *N. benthamiana*

After enzymatic digestion, the target fragment from the fusion entry vector pDONR221-*ScWRKY5* was subjected to the overexpression vector pEarleyGate 203 by the Gateway LR Clonase II Enzyme mix (Invitrogen). After PCR verification, sequencing, and plasmid digestion, the correct recombinant overexpression vector pEarleyGate 203-*ScWRKY5* was obtained. The empty vector pEarleyGate 203 and the recombinant vector pEarleyGate 203-*ScWRKY5* were transformed into *Agrobacterium* GV3101. The *Agrobacterium* strain GV3101 carrying the recombinant plasmid was cultured in an Luria–Bertani (LB) liquid medium containing 50 μg/mL kanamycin and 35 μg/mL rifampicin at 200 rpm and 28 °C. *Agrobacterium* cells cultured overnight were collected by centrifugation, resuspended in MS liquid medium containing 200 μM acetylsyringone to OD_600_ = 0.8, and then the *Agrobacterium* cells were injected into 8-leaf-stage *N. benthamiana* leaves on the plate. The control was the *Agrobacterium* strain GV3101 cell carrying the pEarleyGate 203 empty vector^73,74^. After one day, the leaves were collected for qRT-PCR and DAB histochemical analysis. The tobacco bacterial pathogen *R. solanacearum* and fungal pathogen *F. solani* var. *coeruleum* were cultivated in potato dextrose water (PDW), diluted to OD_600_ = 0.5 with 10 mM magnesium chloride (MgCl_2_) solution, and inoculated into the overexpressing *N. benthamiana* leaves for one day. All of the treated plants were kept for a week under 28 °C with a light period of 16 h/8 h of darkness to track the changes in leaf symptoms and analyze the relative transcription levels of nine tobacco immune-related marker genes. The qRT-PCR was used to analyze the expression levels of the target gene and some tobacco immune marker-related genes in *N. benthamiana*, including the HR marker genes *NtHSR201*, *NtHSR203* and *NtHSR515*, SA-related gene *NtPR-1a/c*, *NtPR2* and *NtPR3*, and ET synthesis-dependent genes *NtEFE26* and *NtAccdeaminase* (Table S2)^78,79^. *NtEF1-α* (GenBank Accession No. D63396) was used as an internal reference (Table S2)^78,79^.

## Conclusions

**Figure 9:**
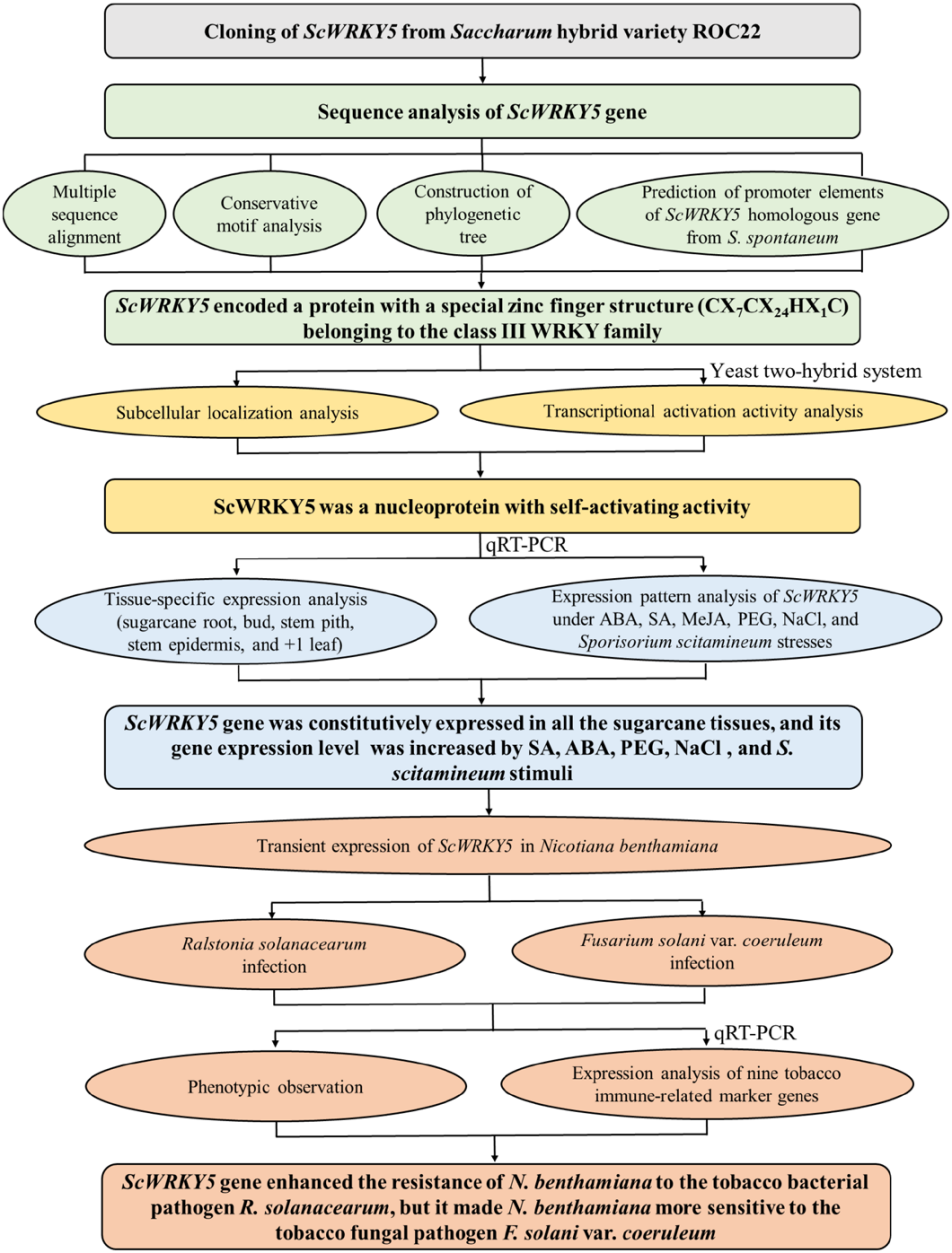
The mechanism-flow chart of the potential function of *ScWRKY5*.

In the present study, a *ScWRKY5* gene was isolated from sugarcane ROC22 and was found to encode a protein with a special zinc finger structure belonging to the class III WRKY family. ScWRKY5 was a nucleoprotein with self-activating activity. Through the prediction of the upstream promoter sequence of the *S. spontaneum WRKY* (Sspon.03g0003780-2c), as a homologue gene of *ScWRKY5*, several *cis*-acting elements involved in photosystem, hormone signaling substances, and abiotic stresses were found. The *ScWRKY5* gene was constitutively expressed in different sugarcane tissues, and its expression level was increased under the stresses of SA, ABA, PEG, and NaCl. After infected by *S. scitamineum* for 1 d, the expression level of the *ScWRKY5* gene was increased in two smut-resistant varieties (YZ01-1413 and LC05-136), while it was decreased in three smut-susceptible varieties (ROC22, YZ03-103, and FN40). Transient overexpression of the *ScWRKY5* gene enhanced the defense ability of *N. benthamiana* to the tobacco bacterial pathogen *R. solanacearum*, but reduced the resistance response to the tobacco fungal pathogen *F*. *solani* var. *coeruleum.* Here, the potential function of *ScWRKY5* in sugarcane was showed in a mechanism-flow chart (Fig. 9). These results may provide references for the excavation and functional identification of *ScWRKY* family genes in sugarcane.

## Supplementary information

Supplementary Information.
