# Supplementary material for: A class III WRKY transcription factor in sugarcane was involved in biotic and abiotic stress responses
Source: Sci Rep. 2020 Dec 1;10:20964. doi: 10.1038/s41598-020-78007-9 (PMC7708483; doi:10.1038/s41598-020-78007-9)
Supplement: Supplementary file 1 — Supplementary Information. [file 41598_2020_78007_MOESM1_ESM.docx]

**A class III WRKY transcription factor in sugarcane was involved in biotic and abiotic stress responses**

**Dongjiao Wang^1^, Ling Wang^1^, Weihua Su^1^, Yongjuan Ren^1^, Chuihuai You^1,2^, Chang Zhang^1^, Youxiong Que^1,3*^ & Yachun Su****^1,3*^**

^1^Key Laboratory of Sugarcane Biology and Genetic Breeding, Ministry of Agriculture, College of Agriculture, Fujian Agriculture and Forestry University, Fuzhou 350002, China. ^2^College of Life Sciences, Fujian Agriculture and Forestry University, Fuzhou 350002, Fujian, China. ^3^ Key Laboratory of Genetics, Breeding and Multiple Utilization of Crops, Ministry of Education, College of Agriculture, Fujian Agriculture and Forestry University, Fuzhou 350002, Fujian, China. *e-mail: queyouxiong@126.com; syc2009mail@163.com

**Additional information**


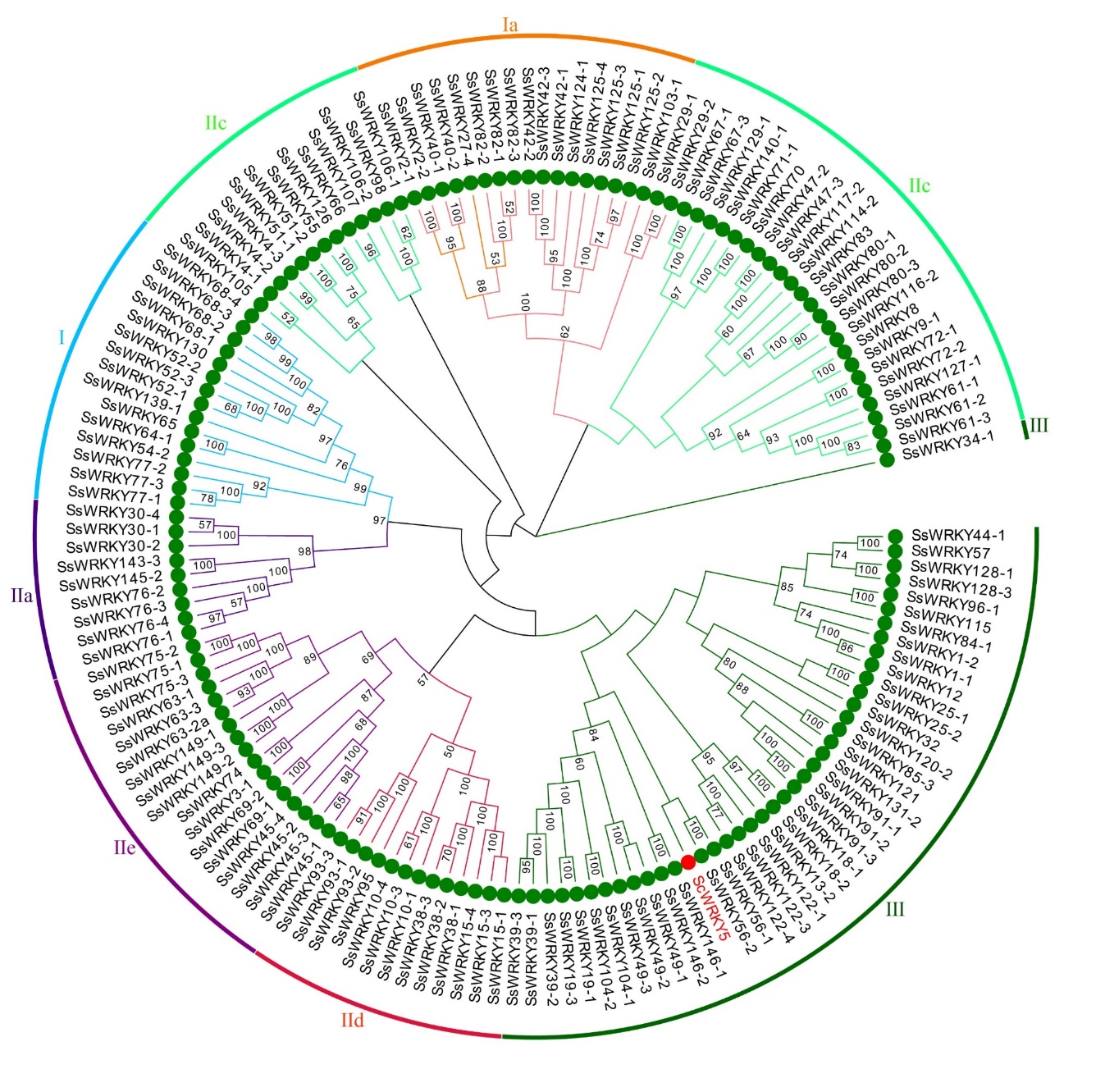


**Figure S1.** Phylogenetic tree analysis of the ScWRKY5 protein and WRKYs from *Saccharum spontaneum*. SsWRKY, *S. spontaneum* WRKY. The phylogenetic tree was plotted using the neighbor-joining (NJ) method with 1000 bootstrap replicates in MEGA 7.0 software. Different colors represent different groups. The ScWRKY5 protein is marked by a red circle.


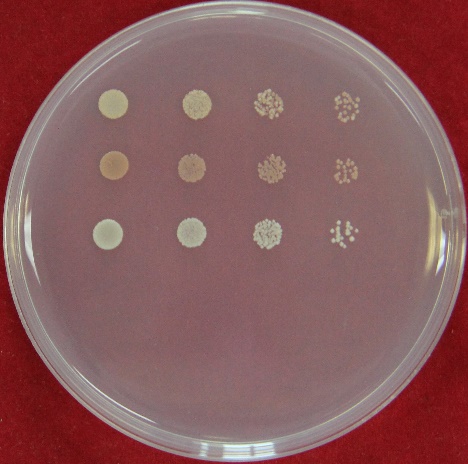

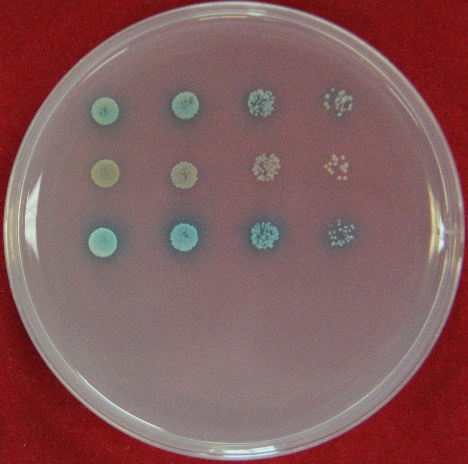

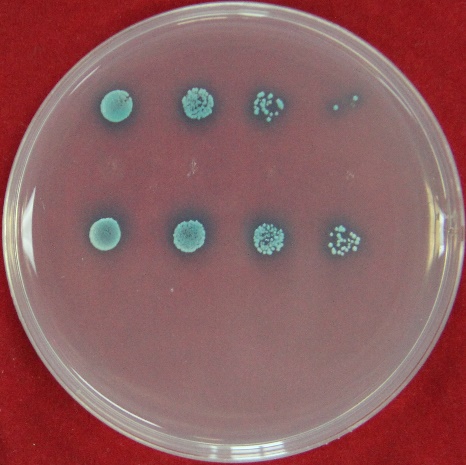


**Positive control**

**pGADT7-T + pGBKT7-p53**

**Negative control**

**pGBKT7**

**pGBKT7-*ScWRKY5***

**10^-1^  10^-2^ 10^-3^ 10^-4^**

**SDO**

**SDO/X**

**SDO/X/A**

**10^-1^  10^-2^ 10^-3^ 10^-4^**

**10^-1^  10^-2^ 10^-3^ 10^-4^**

**Figure S2.** The raw images of the ScWRKY5 transactivation activity test. SDO (SD/-Trp), synthetic dropout medium without tryptophan; SDO/X (SD/-Trp/X-α-Gal), synthetic dropout medium without tryptophan, but plus 5-bromo-4-chloro-3-indoxyl-α-D-galactopyranoside; SDO/X/A (SD/-Trp/X-α-Gal/AbA), synthetic dropout medium without tryptophan but plus 5-bromo-4-chloro-3-indoxyl-α-D-galactopyranoside and aureobasidin A. pGADT7-T + pGBKT7-p53, positive control; pGBKT7, negative control.


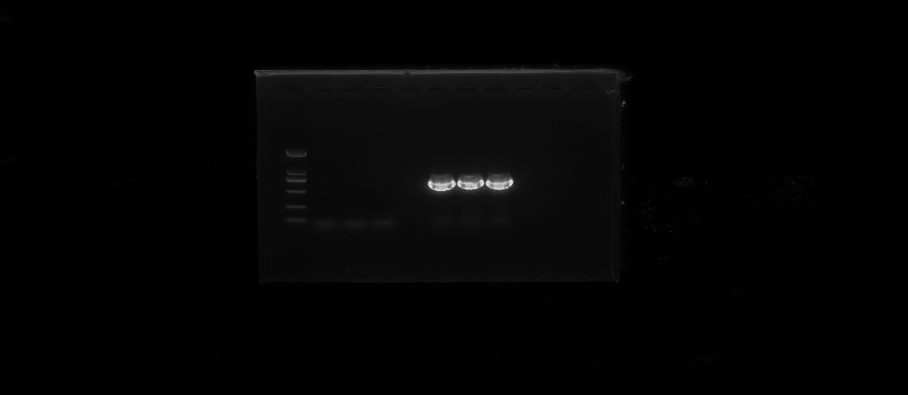


**2000 bp**

**1500 bp**

**750 bp**

**500 bp**

**250 bp**

**100 bp**


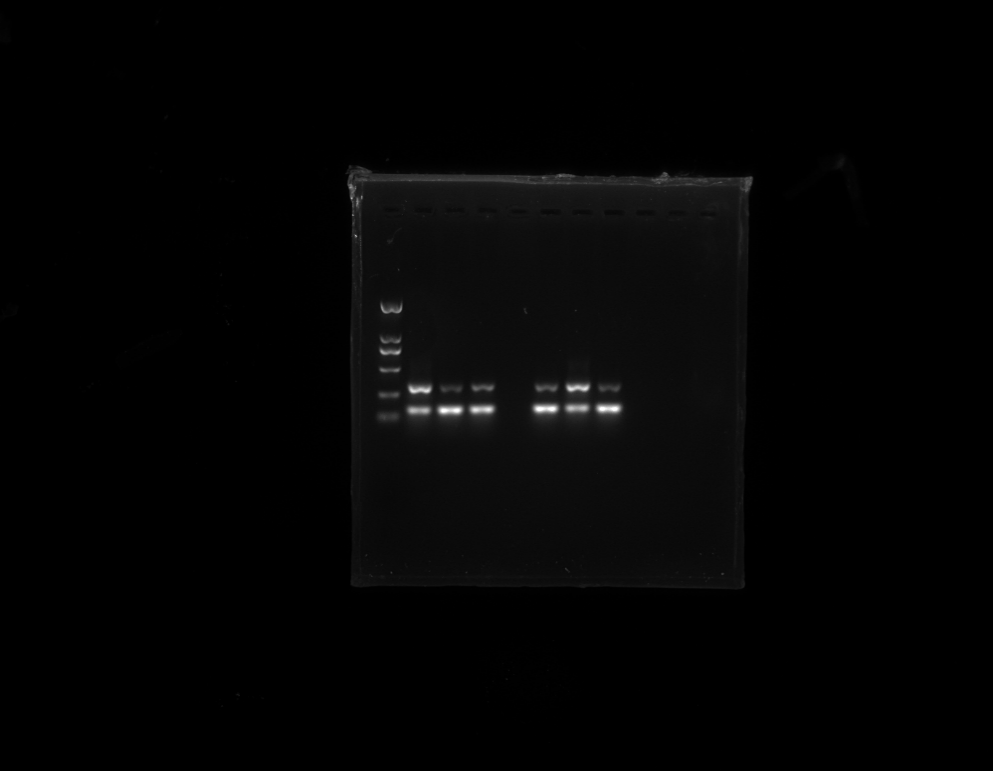


**2000 bp**

**1500 bp**

**750 bp**

**500 bp**

**250 bp**

**100 bp**

**A**

**B**

**Figure S3.** The raw images of the semi-quantitative PCR analysis of *ScWRKY5* in *N. benthamiana* leaves inoculated with *Agrobacterium* GV3101 carrying pEarleyGate 203-*ScWRKY5* (*35S::ScWRKY5*) and the empty vector pEarleyGate 203 (*35S::00*). (A) The semi-quantitative PCR analysis of *ScWRKY5* gene. (B) The semi-quantitative PCR analysis of *NtEF-1α* gene. The target amplified fragments in *35S::00* and *35S::ScWRKY5* samples are marked by a blue and purple rectangles, respectively.

**Table S1.** The *cis*-elements in the promoter of *Saccharum spontaneum* *WRKY* (Sspon.03g0003780-2c)

| **Name** | **Sequence** | **Position** | **No.** | **Function** |
| --- | --- | --- | --- | --- |
| TGACG-motif | TGACG | 613-, 941-, 1116+, 1455- | 4 | *cis*-acting regulatory element involved in the MeJA-responsiveness |
| CGTCA-motif | CGTCA | 613+, 941+, 1116-, 1455+ | 4 | *cis*-acting regulatory element involved in the MeJA-responsiveness |
| GA-motif | ATAGATAA | 1325- | 1 | part of a light responsive element |
| GATA-motif | GATAGGA | 1235- | 1 | part of a light responsive element |
| 4cl-CMA2b | TCTCACCAACCACA | 51+ | 1 | light responsive element |
| GT1-motif | GGTTAA | 1027-, 1108- | 2 | light responsive element |
| G-box | TACGTG | 1088- | 1 | *cis*-acting regulatory element involved in light responsiveness |
| G-Box | CACGTT | 1667+ | 1 | *cis*-acting regulatory element involved in light responsiveness |
| Box 4 | ATTAAT | 94+, 631+, 778+ | 3 | part of a conserved DNA module involved in light responsiveness |
| AE-box | AGAAACAA | 153- | 1 | part of a module for light response |
| P-box | CCTTTTG | 23-, 1241- | 2 | gibberellin-responsive element |
| MBS | CAACTG | 667-, 1405+ | 2 | MYB binding site involved in drought-inducibility |
| RY-element | CATGCATG | 1658- | 1 | *cis*-acting regulatory element involved in seed-specific regulation |
| TATA-box | ATATAT/TATATA/TATA/TATAA/TATACA/TATTTAAA/ccTATAAAaa/TACATAAA | 88+, 89+, 90+, 91+, 179+, 322-, 323+, 419+, 496+, 572-, 574+, 641+, 935+, 997+, 999+, 1236+, 1350-, 1351-, 1352-, 1353-, 1481-, 1482-, 1483-, 1595+ | 24 | core promoter element around -30 of transcription start |
| MBSI | aaaAaaC(G/C)GTTA | 1710+ | 1 | MYB binding site involved in flavonoid biosynthetic genes regulation |
| GCN4_motif | TGAGTCA | 1379- | 1 | *cis*-regulatory element involved in endosperm expression |
| ARE | AAACCA | 326+ | 1 | *cis*-acting regulatory element essential for the anaerobic induction |
| TCA-element | CCATCTTTTT | 716+ | 1 | *cis*-acting element involved in salicylic acid responsiveness |
| ABRE | ACGTG | 1088-, 1665-, 1667- | 3 | *cis*-acting element involved in the abscisic acid responsiveness |
| CAAT-box | CAAAT/CAAT/CCAAT/CAAAT/CAACCAACTCC | 148-, 303+, 512+, 599+, 694-, 914+, 953-, 1044-, 1306+, 1440-, 1721-, 1765+, 1897-, 1923- | 14 | common cis-acting element in promoter and enhancer regions |

+, Forward sequence; -, Reverse sequence.

**Table S2.** Primers used in this study

| **Primer name** | **Forward primer (5’-3’)** | **Usage** |
| --- | --- | --- |
| ScWRKY5-F | CCGGTCGTCGTCAAACCATA | Full length amplification |
| ScWRKY5-R | TTCTGGCAGTGCATGTCCTA | Full length amplification |
| ScWRKY5-QF | TTGCTGCCTCTGGACATACG | qRT-PCR analysis |
| ScWRKY5-QR | TCGAAGGACTAGCCGCATTG | qRT-PCR analysis |
| GAPDH-F | CACGGCCACTGGAAGCA | qRT-PCR analysis |
| GAPDH-R | TCCTCAGGGTTCCTGATGCC | qRT-PCR analysis |
| ScWRKY5-Gate-F | GGGGACAAGTTTGTACAAAAAAGCAGGCTTCATGGCTAAGAGGGATGACTA | Gateway entry vector construction  and semi-quantitative PCR analysis |
| ScWRKY5-Gate-R | GGGGACCACTTTGTACAAGAAAGCTGGGTCTTCTATGAACCAACCCAAAT | Gateway entry vector construction  and semi-quantitative PCR analysis |
| ScWRKY5-BD-F | GGAATTCCATATGATGGCTAAGAGGGATGACTA | Bait vector construction |
| ScWRKY5-BD-R | CCGGAATTCTTCTATGAACCAACCCAAAT | Bait vector construction |
| NtH201-F | CAGCAGTCCTTTGGCGTTGTC | qRT-PCR analysis |
| NtH201-R | GCTCAGTTTAGCCGCAGTTGTG | qRT-PCR analysis |
| NtH203-F | TGGCTCAACGATTACGCA | qRT-PCR analysis |
| NtH203-R | GCACGAAACCTGGATGG | qRT-PCR analysis |
| NtH515-F | TTGGGCAGAATAGATGGGTA | qRT-PCR analysis |
| NtH515-R | TTTGGTGAAAGTCTTGGCTC | qRT-PCR analysis |
| NtNPR1-F | GGCGAGGAGTCCGTTCTTTAA | qRT-PCR analysis |
| NtNPR1-R | TCAACCAGGAATGCCACAGC | qRT-PCR analysis |
| NtPR1a/c-F | AACCTTTGACCTGGGACGAC | qRT-PCR analysis |
| NtPR1a/c-R | GCACATCCAACACGAACCGA | qRT-PCR analysis |
| NtPR2-F | TGATGCCCTTTTGGATTCTATG | qRT-PCR analysis |
| NtPR2-R | AGTTCCTGCCCCGCTTT | qRT-PCR analysis |
| NtPR3-F | CAGGAGGGTATTGCTTTGTTAGG | qRT-PCR analysis |
| NtPR3-R | CGTGGGAAGATGGCTTGTTGTC | qRT-PCR analysis |
| NtEFE26-F | CGGACGCTGGTGGCATAAT | qRT-PCR analysis |
| NtEFE26-R | CAACAAGAGCTGGTGCTGGATA | qRT-PCR analysis |
| NtAccdeaminase-F | TCTGAGGTTACTGATTTGGATTGG | qRT-PCR analysis |
| NtAccdeaminase-R | TGGACATGGTGGATAGTTGCT | qRT-PCR analysis |
| NtEF-1α-F | TGCTGCTGTAACAAGATGGATGC | qRT-PCR analysis |
| NtEF-1α-R | GAGATGGGGACAAAGGGGATT | qRT-PCR analysis |

*attB1* and *attB2* adapters are underlined in the primers of ScWRKY5-Gate-F and ScWRKY5-Gate-R for the construction of gateway entry vector, respectively. The added restriction enzyme sites of *Nde* I (CATATG) and *Eco*R I (GAATTC) are underlined in the primers of ScWRKY5-BD-F and ScWRKY5-BD-R for the construction of bait vector, and the double underlined in these primers indicate protective bases.
